# Fatty acid composition in serum cholesterol esters and phospholipids is linked to visceral and subcutaneous adipose tissue content in elderly individuals: a cross-sectional study

**DOI:** 10.1186/s12944-017-0445-2

**Published:** 2017-04-04

**Authors:** Fredrik Rosqvist, Helena Bjermo, Joel Kullberg, Lars Johansson, Karl Michaëlsson, Håkan Ahlström, Lars Lind, Ulf Risérus

**Affiliations:** 1 Department of Public Health and Caring Sciences, Clinical Nutrition and Metabolism, Uppsala University, Uppsala, Sweden; 2 Department of Radiology, Uppsala University Hospital, Uppsala University, Uppsala, Sweden; 3 Department of Surgical Sciences, Section of Orthopaedics, Uppsala University, Uppsala, Sweden; 4 Department of Medical Sciences, Uppsala University Hospital, Uppsala University, Uppsala, Sweden

**Keywords:** Visceral adipose tissue, Polyunsaturated fat, Saturated fat, Linoleic acid, Palmitic acid, Adipose tissue distribution, Fatty acid, Body fat

## Abstract

**Background:**

Visceral adipose tissue (VAT) and truncal fat predict cardiometabolic disease. Intervention trials suggest that saturated fatty acids (SFA), e.g. palmitic acid, promote abdominal and liver fat storage whereas polyunsaturated fatty acids (PUFA), e.g. linoleic acid, prevent fat accumulation. Such findings require investigation in population-based studies of older individuals. We aimed to investigate the relationships of serum biomarkers of PUFA intake as well as serum levels of palmitic acid, with abdominal and total adipose tissue content.

**Methods:**

In a population-based sample of 287 elderly subjects in the PIVUS cohort, we assessed fatty acid composition in serum cholesterol esters (CE) and phospholipids (PL) by gas chromatography and the amount of VAT and abdominal subcutaneous (SAT) adipose tissue by magnetic resonance imaging (MRI), liver fat by MR spectroscopy (MRS), and total body fat, trunk fat and leg fat by dual-energy X-ray absorptiometry (DXA). Insulin resistance was estimated by HOMA-IR.

**Results:**

VAT and trunk fat showed the strongest correlation with insulin resistance (*r* = 0.49, *P* < 0.001). Linoleic acid in both CE and PL was inversely related to all body fat depots (*r* = −0.24 to −0.33, *P* < 0.001) including liver fat measured in a sub-group (*r* = −0.26, *P* < 0.05, *n* = 73), whereas n-3 PUFA showed weak inverse (18:3n-3) or positive (20:5n-3) associations. Palmitic acid in CE, but not in PL, was directly correlated with VAT (*r* = 0.19, *P* < 0.001) and trunk fat (*r* = 0.18, *P* = 0.003). Overall, the significant associations remained after adjusting for energy intake, height, alcohol, sex, smoking, education and physical activity. The inverse correlation between linoleic acid and VAT remained significant after further adjustment for total body fat.

**Conclusions:**

Serum linoleic acid is inversely related to body fat storage including VAT and trunk fat whereas palmitic acid was less consistently but directly associated, in line with recent feeding studies. Considering the close link between VAT and insulin resistance, a potential preventive role of plant-based PUFA in VAT accumulation warrants further study.

## Background

While obesity is associated with metabolic disorders, the relative accumulation of both visceral adipose tissue (VAT) and abdominal subcutaneous adipose tissue (SAT) is probably of greater importance than the total amount of adipose tissue [1] . In particular, VAT has been linked with insulin resistance [2–4], although little is known about factors determining VAT deposition. The role of diet for body fat distribution is poorly understood, but accumulating data suggest that differences in dietary fat composition are involved [5–7]. Animal studies have indicated that diets rich in polyunsaturated fatty acids (PUFA) result in lower fat accumulation [8–11] compared with saturated fatty acids (SFA), possibly through greater fat oxidation compared with long-chain SFA [12–14]. Furthermore, linoleic acid, 18:2n-6 (LA), may improve insulin sensitivity [7, 15–19], possibly in part through a PUFA-dependent reduction of VAT and liver fat [5, 6]. Compared with PUFA, SFA may also induce lipogenic enzymes such as stearoyl-CoA desaturase (SCD) [20]. Randomized feeding studies have shown that LA, the major dietary PUFA, decrease trunk fat [21], VAT/SAT ratio [6] and abdominal fat [7] compared with a diet high in SFA. Notably, we recently showed that high intake of palmitic acid, 16:0 (PA), for 7 weeks markedly increased VAT, liver fat and total fat accumulation in healthy, young subjects, whereas high intake of LA caused a several-fold lower accumulation of VAT and liver fat [5]. It would be intriguing if such short-term effects could be confirmed in observational settings, potentially reflecting long-term relationships between specific fatty acid intake and body fat distribution. A small study of 24 overweight Japanese men reported a direct association between VAT thickness and serum PA, and an inverse association with LA [22]. Furthermore, cross-sectional analyses in a large population-based sample (*n* = 3926) observed higher prevalence of abdominal obesity in subjects with higher serum PA and lower serum LA [23].

Altered fatty acid desaturase activities may also be involved in body fat accumulation, e.g. deletion of SCD activity reduces fat mass in animals [24]. Estimated hepatic SCD activity was decreased by PUFA, possibly mediating the reduced fat accumulation [5, 6].

Fatty acid composition in cholesterol esters (CE) is an objective marker of dietary intake, and intake of PUFA such as LA in particular is reflected well in serum CE [25–28] and correlates well with self-reported fatty acid intake in Swedish [29] and other cohorts [27]. Intake of major SFA such as PA is also reflected in serum CE, but to a lesser extent than PUFA due to endogenous synthesis and further elongation [17].

In a population-based sample of elderly individuals we aimed to investigate the associations between serum fatty acids, especially those used in feeding trials (i.e. PA and LA, as well as n-3 PUFA, which are valid dietary biomarkers), and adipose tissue content assessed by magnetic resonance imaging (MRI) and dual-energy X-ray absorptiometry (DXA). Furthermore we examined associations between body fat distribution with estimated desaturase activities and with insulin resistance, respectively.

## Methods

Subjects participated in the Prospective Investigation of the Vasculature in Uppsala Seniors (PIVUS) cohort study. All individuals who were 70 years of age and living in Uppsala, Sweden, were invited. Between April 2001 and June 2004, 2,025 subjects were randomly invited within one month of their seventieth birthday in order to standardize for age, and 1,016 (50.1%) chose to participate. MRI was performed on 287 randomly selected subjects, which represents the current study population. All participants gave written informed consent and the study was approved by the regional ethical review board in Uppsala. Blood samples and anthropometric measurements were taken after an overnight fast. Body mass index (BMI) was calculated as weight (kg) divided by height (m) squared. Insulin resistance was assessed by homeostasis model of assessment insulin resistance (HOMA-IR) [30].

### Assessment of fatty acid composition

Fatty acid composition in serum cholesterol esters (CE) and phospholipids (PL) were measured by gas chromatography. Serum (0.5 mL) was mixed with 2.5 mL methanol, 5 mL chloroform (with 0.005% added butylated hydroxytoluene, BHT) and 7.5 mL NaH_2_PO_4_ (0.2 mol/l) and stored at 4 °C overnight for lipid extraction. The chloroform phase was then removed with a syringe and evaporated to dryness on a 30 °C heating block using nitrogen gas. The lipid residue was dissolved in chloroform and the lipid esters were separated by thin-layer chromatography (TLC); the adsorbent containing POPOP was used as fluorescent agent. The TLC plates were eluted at room temperature with the solvent system petroleum ether/diethyl ether/acetic acid (81:18:1 by volume). The lipid fractions were visualized in UV light; the spots containing cholesterol esters and phospholipids were scraped off into vials and the lipid esters were then methylated at 60 °C overnight after addition of 2 mL H_2_SO_4_ (5%) methanol. The fatty acid methyl esters were extracted into 3 mL petroleum ether (0.005% BHT) after addition of 1.5 mL distilled water. The phases were separated after thorough mixing and centrifugation at 1500 g for 10 min. The petroleum ether phase was pipetted off and the solvent was evaporated under nitrogen gas on a 30 °C heating block. The fatty acid methyl esters were dissolved in 120 μL hexane and placed in vials. The fatty acid methyl esters were separated by gas–liquid chromatography on a 30-m glass capillary column coated with Thermo TR-FAME (Thermo Electron Corporation, USA) with helium gas as a carrier gas. An Agilent Technologies system consisting of model GLC 6890 N, autosampler 7683 and Agilent ChemStation was used. The temperature was programmed to 150–260 °C. The fatty acids were identified by comparing each peak’s retention time with fatty acid methyl ester standards Nu Check Prep (Elysian, MN, USA). In 20 replicates, the CV% for included fatty acids was 0.37–2.49 in CE and 0.52–1.27 in PL. Fatty acids are presented as the relative sum of the fatty acids analysed. Desaturase activities were estimated as product-to-precursor ratios of individual fatty acids as follows: SCD; 16:1*n*-7/16:0, delta-5 desaturase; 20:4n–6/20:3n-6 and delta-6 desaturase; 18:3n–6/18:2n–6.

### Assessment of body fat content and distribution

VAT and SAT were measured by MRI. MRI was performed using a single axial 10 mm slice at the L4-L5 interface. VAT and SAT areas (cm^2^) were assessed using the software package ImageJ by manual contouring of the two tissues. The methodology has previously been described [31]. Based on repeated measurements in 22 of the subjects, the CVs of VAT and SAT were found to be 5.9 and 3.4%, respectively. Total body fat, trunk fat and leg fat were measured by DXA. By triple measurements in 15 subjects, the precision error of the DXA measurements (DPX Prodigy, Lunar corp., Madison, WI, USA) was 1.5% for total fat mass. In a subsample of *n* = 73 subjects from the whole PIVUS cohort, liver fat was measured using a single volume 1H-spectroscopy acquisition that was performed with and without water-suppression (WS) during shallow breathing. A volume of interest 3x3x3 cm was positioned in the right lobe of the liver and data were obtained using TR/TE = 3000/30 ms with 16 excitations without WS and 64 with WS. A single volume 1H-spectroscopy acquisition was performed with and without WS. All spectroscopy analyses were performed with the MRUI software (version 2.2) using water as an internal reference giving intrahepatocellular lipid levels as output in %. DXA and MRI measurements were performed in the non-fasted state, on average two years after the baseline investigation at age 70.

### Assessment of physical activity and dietary intake

Physical activity was assessed by asking the participant how many times per week he/she performed light (e.g. walking, gardening) and hard exercise (e.g. running, swimming) for at least 30 min. A 7-day pre-coded dietary record was used to assess energy intake.

### Statistical analyses

The distribution of the variables was examined by the Shapiro-Wilk W test. To attain normal distribution, 20:5n-3 (EPA), SCD, delta-6 desaturase, SAT, leg fat, VAT and HOMA-IR were log-transformed. The correlation between serum fatty acids and adipose tissue content was investigated by Pearson’s correlation analysis. The Benjamini-Hochberg procedure was used to correct for multiple comparisons. Multiple regression analyses were performed with energy intake, height, alcohol intake, sex, smoking, education and physical activity as covariates. Collinearity among included covariates was assessed by the variance inflation factor (VIF). A *p*-value <0.05 was considered statistically significant. Based on the current sample size, we had the ability to detect correlations stronger than ~0.16 with α = 0.05 and β = 0.80 (R code: pwr.r.test(*n* = 287, *r*= , sig.level = 0.05, power = .8). JMP software version 10.0.0 was used for statistics (SAS Institute, Inc).

## Results

### Baseline characteristics

As a group, this elderly population was overweight but generally healthy (Table 1). Men (*n* = 148, 52%) and women (*n* = 139, 48%) were equally represented in the population. Based on the pre-coded dietary record, subjects had an energy intake of 1887 ± 467 kcal/day (energy percent from protein, carbohydrates, fat and alcohol were 16.3, 48.9, 31.1 and 2.0, respectively). The median number of sessions (30 min) of light exercise per week was 3 (IQR 2 to 6) and the median number of sessions (30 min) of hard exercise per week was 0 (IQR 0 to 1).

**Table 1:** Baseline characteristics

Women/Men (%)	48/52
BMI (kg/m2)	26.8 ± 4.1
Visceral Adipose Tissue (cm^2^)	96.2 (64.9 to 138.8)
Subcutaneous Adipose Tissue (cm^2^)	208.8 (151.3 to 283.2)
Total Body Fat (kg)	25.1 ± 8.5
Trunk Fat (kg)	13.8 ± 5.0
Leg Fat (kg)	7.6 (5.4 to 9.7)
Waist circumference (cm)	90.7 ± 11.1
Waist/hip ratio	0.9 ± 0.1
Glucose (mM)	5.0 (4.6 to 5.5)
Insulin (pM)	50.0 (36.1 to 72.9)
Insulin resistance (HOMA-IR)	1.6 (1.1 to 2.5)
Systolic Blood Pressure (mmHg)	146 ± 19
Diastolic Blood Pressure (mmHg)	69 ± 8
Total cholesterol (mM)	5.4 ± 1.0
HDL cholesterol (mM)	1.5 ± 0.4
LDL cholesterol (mM)	3.3 ± 0.8
Triacylglycerols (mM)	1.2 (0.9 to 1.6)
Serum fatty acids (CE)^a^	
16:0 (PA) (%)	12.0 ± 0.8
18:2n-6 (LA) (%)	47.6 ± 4.2
18:3n-3 (ALA) (%)	0.9 ± 0.2
20:5n-3 (EPA) (%)	2.1 (1.6 to 2.9)
22:6n-3 (DHA) (%)	1.0 ± 0.3

Data are presented as mean (SD) or median (IQR).^a^Data are percentage of the total fatty acids analysed. Triacylglycerols, total cholesterol, HDL cholesterol and insulin were analyzed in serum (LDL cholesterol was calculated according to the Friedewalds formula). Glucose was analysed in plasma

### Serum fatty acids, body fat distribution and liver fat

**Fig. 1:**
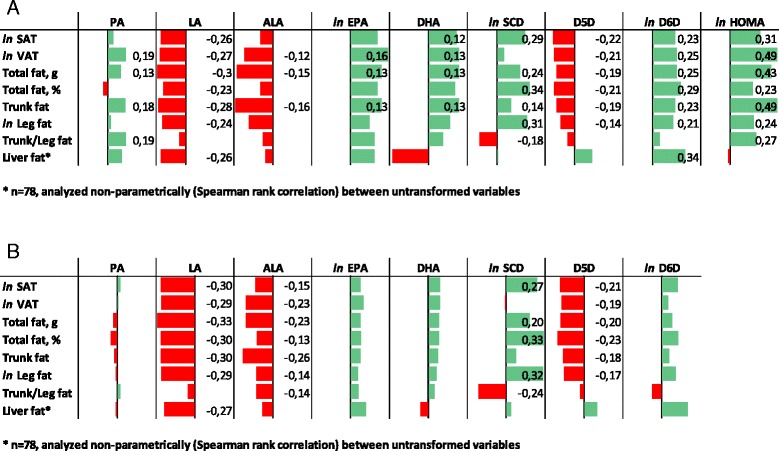
Correlations between serum fatty acids and desaturase indices in serum cholesterol esters (**a**) and serum phospholipids (**b**) and adipose tissue depots. Bars are Pearson correlation coefficient (r). The correlation coefficient is given as a number beside the bar for all significant correlations (*P* < 0.05). Abbreviations: PA, palmitic acid; LA, linoleic acid; ALA, alpha-linoleic acid; EPA, eicosapentaenoic acid; DHA, docosahexaenoic acid; SAT, subcutaneous adipose tissue; VAT, visceral adipose tissue; SCD, stearoyl-CoA desaturase; D5D, delta-5 desaturase; D6D, delta-6 desaturase; HOMA, homeostasis model of assessment insulin resistance

In serum cholesterol esters, PA was directly associated with VAT, trunk fat, total fat and the trunk-to-leg fat ratio, but not with SAT or percent body fat (Fig. 1a). Conversely, LA was inversely related to SAT, VAT, trunk fat, leg fat, total fat and percent body fat. The vegetable n-3 PUFA 18:3n-3 (ALA) was negatively associated with VAT, trunk fat and total fat, whereas the marine n-3 PUFAs 20:5n-3 (EPA) and 22:6n-3 (DHA) were positively correlated with fat depots. Importantly, when the correlations between EPA and DHA with fat depots were adjusted for LA, none of the correlations remained statistically significant (Table 2). In the subsample with liver fat measurement, LA was inversely associated with liver fat content (rho = −0.26, P = 0.028). The majority of correlations remained significant after correction for multiple testing with false discovery rate (FDR) set at 5%, and all correlations were significant at FDR 7%. Most associations remained significant in the multivariate model (Table 3). When comparing subjects in the extreme deciles of plasma LA, the differences in total fat, SAT and VAT were 9 kg, 62 cm^2^ and 52 cm^2^, respectively. Overall, the significant associations did not seem to be clearly sex-specific, although the correlation between PA and SAT was stronger in men than in women, and the association between delta-5 desaturase activity index and all fat depots were stronger in women than in men (data not shown).

**Table 2:** Multiple regression analyses between EPA and DHA with fat depots, with LA as a covariate

	*ln* EPA	DHA
*ln* SAT		
β	−0.02	0.07
P	0.78	0.51
*ln* VAT		
β	0.04	0.10
P	0.60	0.46
Total fat, g		
β	−430.2	812.8
P	0.73	0.68
Total fat, %		
β	0.09	1.03
P	0.94	0.60
Trunk/Leg fat		
β	0.13	0.11
P	0.18	0.48
Trunk fat		
β	−65.7	634.6
P	0.93	0.59
*ln* Leg fat		
β	−0.04	0.02
P	0.54	0.87

*SAT*, abdominal subcutaneous adipose tissue; *VAT*, visceral adipose tissue; *EPA*, eicosapentaenoic acid; *DHA*, docosahexaenoic acid

**Table 3:** Fatty acids and desaturases in cholesterol esters in relation to body fat depots

	PA	LA	ALA	*ln* EPA	DHA	*ln* SCD	D5D	*ln* D6D
*ln* SAT								
β	0.08	−0.03	−0.3	0.12	0.18	0.35	−0.04	0.21
P	0.04	<0.01	0.03	0.06	0.10	<0.01	<0.01	<0.01
R2	0.21	0.26	0.22	0.21	0.21	0.24	0.23	0.24
pR2	0.002	0.07	0.003	0.01	0.01	0.08	0.05	0.05
*ln* VAT								
β	0.12	−0.04	−0.4	0.29	0.39	0.24	−0.05	0.34
P	0.02	<0.01	0.03	<0.01	<0.01	0.07	<0.01	<0.01
R2	0.18	0.24	0.18	0.20	0.19	0.17	0.19	0.22
pR2	0.04	0.07	0.01	0.02	0.02	0.004	0.05	0.06
Total fat, g								
β	1645.9	−581.7	−7598.1	2582.7	3917.5	5955.6	−663.6	4001.2
P	0.03	<0.01	<0.01	0.03	<0.05	<0.01	0.01	<0.01
R2	0.17	0.23	0.19	0.17	0.17	0.19	0.18	0.20
pR2	0.02	0.09	0.02	0.02	0.02	0.06	0.03	0.06
Total fat, %								
β	0.95	−0.43	−5.87	2.44	3.10	5.07	−0.58	3.81
P	0.11	<0.01	<0.01	<0.01	0.05	<0.01	<0.01	<0.01
R2	0.43	0.46	0.44	0.44	0.43	0.45	0.44	0.46
pR2	0.002	0.05	0.002	0.01	0.01	0.11	0.04	0.08
Trunk fat, g								
β	942.0	−338.4	−4356.4	1629.9	2615.1	3004.4	−487.8	2429.9
P	0.03	<0.01	<0.01	0.02	0.03	<0.01	<0.01	<0.01
R2	0.16	0.21	0.18	0.17	0.17	0.17	0.18	0.20
pR2	0.03	0.08	0.03	0.02	0.02	0.02	0.04	0.05
*ln* Leg fat								
β	0.07	−0.02	−0.31	0.08	0.11	0.30	−0.02	0.14
P	0.03	<0.01	<0.01	0.10	0.20	<0.01	0.15	<0.01
R2	0.33	0.37	0.34	0.33	0.32	0.36	0.32	0.34
pR2	0.001	0.06	0.01	0.007	0.008	0.10	0.02	0.05
Trunk/Leg fat								
β	0.05	−0.01	−0.03	0.18	0.28	−0.08	−0.04	0.16
P	0.26	0.10	0.86	<0.01	0.02	0.46	<0.01	0.01
R2	0.45	0.45	0.45	0.46	0.46	0.45	0.46	0.46
pR2	0.04	0.003	0.002	0.01	0.004	0.03	0.005	0.005

*SAT*, abdominal subcutaneous adipose tissue; *VAT*, visceral adipose tissue; *SCD1*, stearoyl-CoA desaturase; *D5D*, delta-5 desaturase; *D6D*, delta-6 desaturase; *PA*, palmitic acid; *LA*, linoleic acid; *ALA*, alpha-linoleic acid; *EPA*, eicosapentaenoic acid; *DHA*, docosahexaenoic acid; *pR2*, partial R2

Multiple regression analyses with energy intake, height, alcohol intake, sex, smoking, education and physical activity as covariates

The positive associations between PA and VAT and trunk fat remained statistically significant after adjustment for total body fat, as did the negative association between LA and VAT (data not shown). However, the associations for PA were lost when all covariates were included in the model, but the negative association between LA and VAT still remained statistically significant (data not shown).

In serum phospholipids, the overall pattern of associations was similar to that for cholesterol esters (Fig. 1b), but associations were stronger for LA and ALA but weaker for PA, EPA and DHA. In the multivariate model results were generally similar to that for cholesterol esters (Table 4), but most apparently so for LA, ALA, SCD and D5D, whereas results for PA and D6D lost significance.

**Table 4:** Fatty acids and desaturases in phospholipids in relation to body fat depots

	PA	LA	ALA	*ln* EPA	DHA	*ln* SCD	D5D	*ln* D6D
*ln* SAT								
β	0.02	−0.05	−1.06	0.09	0.03	0.25	−0.11	0.03
P	0.42	<0.01	<0.01	0.19	0.24	0.01	<0.01	0.81
R2	0.20	0.26	0.24	0.20	0.20	0.22	0.22	0.27
pR2	0.0003	0.09	0.02	0.006	0.01	0.07	0.04	0.02
*ln* VAT								
β	0.006	−0.08	−1.57	0.23	0.07	0.12	−0.17	0.02
P	0.85	<0.01	<0.01	<0.01	0.02	0.34	<0.01	0.89
R2	0.17	0.28	0.23	0.19	0.18	0.17	0.20	0.18
pR2	0.0002	0.08	0.05	0.01	0.009	0.00002	0.04	0.002
Total fat, g								
β	34.1	−1063.6	−23269.7	1939.3	605.1	3871.2	−1826.9	331.9
P	0.94	<0.01	<0.01	0.12	0.17	0.04	0.02	0.88
R2	0.15	0.24	0.22	0.16	0.16	0.17	0.17	0.27
pR2	0.0008	0.11	0.05	0.007	0.008	0.04	0.04	0.008
Total fat, %								
β	−0.10	−0.87	−19.4	2.06	0.56	3.25	−1.57	−0.32
P	0.79	<0.01	<0.01	0.04	0.12	0.03	0.01	0.85
R2	0.42	0.48	0.47	0.43	0.42	0.43	0.43	0.52
pR2	0.002	0.09	0.02	0.009	0.007	0.11	0.05	0.02
Trunk fat								
β	−4.55	−639.6	−14518.5	1139.1	435.0	1667.6	−1325.6	220.7
P	0.99	<0.01	<0.01	0.12	0.10	0.13	<0.01	0.86
R2	0.15	0.24	0.22	0.16	0.16	0.16	0.18	0.20
pR2	0.0004	0.09	0.07	0.007	0.007	0.007	0.03	0.004
*ln* Leg fat								
β	0.005	−0.04	−0.78	0.07	0.01	0.22	−0.05	0.02
P	0.82	<0.01	<0.01	0.17	0.60	<0.01	0.17	0.84
R2	0.31	0.37	0.35	0.32	0.31	0.34	0.32	0.41
pR2	0.00004	0.08	0.02	0.004	0.005	0.10	0.03	0.01
Trunk/Leg fat								
β	−0.003	−0.04	−0.89	0.14	0.07	−0.11	−0.11	0.02
P	0.92	<0.01	<0.01	0.06	<0.01	0.31	0.02	0.90
R2	0.46	0.48	0.47	0.47	0.47	0.46	0.47	0.44
pR2	0.0003	0.004	0.02	0.005	0.002	0.06	0.0007	0.006

*SAT*, abdominal subcutaneous adipose tissue; *VAT*, visceral adipose tissue; *SCD1*, stearoyl-CoA desaturase; *D5D*, delta-5 desaturase; *D6D*, delta-6 desaturase; *PA*, palmitic acid; *LA*, linoleic acid; *ALA*, alpha-linoleic acid; *EPA*, eicosapentaenoic acid; *DHA*, docosahexaenoic acid; *pR2*, partial R2

Multiple regression analyses with energy intake, height, alcohol intake, sex, smoking, education and physical activity as covariates

### Desaturation indices and body fat distribution

SCD activity index in serum CE was positively associated with SAT, trunk fat, leg fat and total fat content, but not with VAT (Fig. 1a). Delta-6 desaturase activity index was positively associated with SAT, VAT, trunk fat, leg fat and total fat. Conversely, delta-5 desaturase activity index was inversely associated with all fat depots. Results were similar for serum phospholipids although slightly attenuated (Fig. 1b). Many of the associations remained significant in the multivariate model (Tables 3 and 4).

### Insulin resistance in relation to body fat and fatty acids

Of all adipose tissue depots, VAT and trunk fat clearly showed the strongest association with insulin resistance (*r* = 0.49, *P* < 0.001) for both, although SAT (*r* = 0.31, *P* < 0.001), leg fat (*r* = 0.24, *P* < 0.001), trunk-to-leg fat ratio (*r* = 0.27, *P* < 0.001), percent body fat (*r* = 0.23, *P* < 0.001) and total body fat (*r* = 0.43, *P* < 0.001) were also significantly associated (Fig. 1a).

PA was directly associated (*r* = 0.20, *P* < 0.001) with insulin resistance. Weaker but significant inverse associations were observed for LA (*r* = −0.13, *P* = 0.02) and delta-5 desaturase (*r* = −0.12, *P* = 0.04).

## Discussion

Considering the close association between VAT and insulin resistance, the role of fatty acids as potential modulators of abdominal fat distribution is of interest. In this community-based study we observed that serum PA, partly reflecting dietary intake, was directly associated with VAT volume, whereas serum LA, mainly reflecting dietary n-6 PUFA intake, was inversely associated with VAT, trunk fat, SAT, leg fat, total body fat and percent body fat. Notably, the inverse link between LA and VAT was independent of total body fat, potentially suggesting a rather specific influence of LA on VAT accumulation. Furthermore, LA was inversely associated with liver fat content in a smaller subsample. These results strongly accord with animal studies [8–11], and a double-blinded trial in healthy adults showing that high intake of PA for 7 weeks promotes VAT accumulation, whereas LA prevents VAT, liver- and body fat accumulation [5] instead. This study provides novel evidence that fatty acid-dependent effects on VAT accumulation may also occur long-term, adding to the similar findings in short-term trials. Serum fatty acids in CE partly reflect average intake over the last days to weeks [25, 26, 28] but have shown to be rather stable in Swedish middle-aged and elderly individuals, even over decades [32]. Thus, the present relationships could reflect distinct long-term effects of SFA and PUFA on body fat accumulation. Moreover, the associations may translate to noteworthy effects as supported by several feeding trials [5, 7, 12]. If comparing subjects in the extreme deciles of serum LA, the differences were as much as 9 kg for total body fat, 62 cm^2^ for VAT, and 52 cm^2^ for SAT, respectively. These results also accord with a 5-week randomized study showing that increased dietary PUFA (mainly LA) reduced abdominal SAT compared with dietary SFA (mainly PA) [7]. Furthermore, a controlled study showed that LA-rich vegetable oil reduced trunk adipose mass in women with type 2 diabetes [21], and a randomized study in obese subjects showed reduced VAT/SAT ratio after iso-caloric feeding of LA from sunflower oil versus PA mainly from butter [6]. The current diverse associations between PA and LA with body fat and VAT accord with a small Japanese study of men [22], and with three Scandinavian cohorts reporting inverse relations between LA and BMI, waist circumference and total fat mass [23, 33, 34], but not with a small French study reporting direct associations between LA with BMI and total body fat [35].

The mechanisms behind these observations are unclear, but we recently suggested that SFA and PUFA have diverse effects on certain genes involved in energy dissipation and fat storage, such as aldehyde dehydrogenase 1 family member A1 (ALDH1A1), which was downregulated by LA [5]. It has also been shown that dietary long-chain SFAs (e.g. PA) have slower oxidation [36, 37], and an increase in dietary PA decreased fat oxidation and energy expenditure compared to unsaturated fat [12]. Increased intake of PA over PUFA would thus be expected to increase body fat accumulation over time, as shown in animals [38–40] and humans during 4–7 weeks [6, 14]. An obesity-promoting effect of PA might also be mediated by lower diet-induced thermogenesis [38, 41]. In the present study, PA was positively associated with VAT and total fat volume, but not SAT. This finding accords with our interventional data showing that high intake of PA caused a significant increase in VAT, but not abdominal SAT [5]. Since a significant proportion of PA is desaturated by SCD, the present direct association between estimated hepatic SCD activity and increased SAT, but not VAT, might reflect a protection against a PA-induced enlargement of VAT. In the present observational study, PA in serum CE presumably mainly reflects relative PA content in the liver that is partly derived from dietary PA, but possibly also from de novo lipogenesis during low-fat/high sugar intakes [6]. It should, however, be noted that low fat intakes (<30%E) and high carbohydrate diets were rare among Swedish elderly men 15 years ago, when the data were collected; thus, the contributing role of de novo lipogenesis under such conditions is likely to have a minor determining role of serum PA proportions [42]. Due to the uncertainty about serum PA as a dietary marker of PA intake, the results regarding this fatty acid should be interpreted cautiously. Additionally, the results for PA were not consistent between the two fractions used (CE and PL) whereas LA and ALA, which are better dietary biomarkers, were highly consistent between fractions.

The finding that the main PUFA in the diet and in serum lipids, LA, showed negative associations with VAT, trunk fat, liver fat, SAT, leg fat and total body fat may be due to decreased storage rates of PUFA, and a higher oxidation rate compared with PA [36]. PUFA, such as LA, could also counteract lipogenesis by suppressing lipogenic enzymes [43] and transcription factors [44]. The effects of LA on VAT could potentially be mediated in part through the corticoid system as has been recently suggested [45]. The observation that the estimated SCD activity was directly correlated with SAT and total body fat, but not VAT (although with trunk fat), is partly congruent with previous observational studies in elderly or middle-aged subjects, showing positive associations with fat mass and obesity [23, 33, 34], VAT thickness [22] and to obesity-related diseases [16, 46–48]. Currently, delta-6 desaturase activity was positively associated, whereas delta-5 desaturase activity was inversely associated with all adipose tissue depots, a pattern consistently observed in insulin resistant states and obesity [18, 23, 33, 46].

Surprisingly, the marine n-3 PUFAs EPA and DHA were both positively associated with body fat depots, which is in stark contrast to what has been shown experimentally. These present associations are most likely due to the opposite relationship between the proportions of n-6 and n-3 PUFA. Because they share the same set of enzymes for elongation, high levels of either n-6 or n-3 tends to suppress the other. This interpretation is supported by the fact that all associations between EPA and DHA with fat depots were lost when adjusted for LA. Although fatty acid composition is presented in relative proportions, we believe it is justified to adjust the associations with EPA and DHA for LA due to the abovementioned biological relationship, and because EPA and DHA does not share dietary sources of intake with LA. Failure to consider this may introduce spurious associations.

Notably, VAT (and trunk fat) was the adipose tissue depot most closely associated with insulin resistance (estimated by HOMA-IR), suggesting that diverse influences of fatty acids on VAT may be relevant for metabolic health. Excess VAT could be causally connected to insulin resistance, but might also be a marker of inadequate ability to store excess energy in SAT [49, 50].

Strengths of this study include the population-based sample, that all subjects were of a similar age, and the fact that both exposure and outcomes were assessed with reliable techniques, i.e. gas chromatography was used for assessing serum fatty acids (avoiding bias of self-reported intake) and MRI and DXA for determining body fat content. Based on the current sample size, we had statistical ability to detect the majority (~75%) of all significant correlations. Correcting for multiple comparisons yielded similar results and did not alter the interpretation. Collinearity among covariates in the multiple regression models was not a cause for concern as the maximum VIF value was 2.5 (VIF <10 is generally accepted as low collinearity). The main weakness is the cross-sectional design, thus excluding information about causality. The relatively long lag-time between the MRI measurements and blood sampling may introduce minor errors for some individuals, but is unlikely to alter the results on a group level. Although many of the associations were significant, many of the fatty acids only explained a small degree of the variance in the different body fat depots, implying that other factors (e.g. energy intake) are of greater importance in determining the size of the adipose depots. Thus, the overall pattern of associations are the most relevant in this study, but it is not unlikely that even a relatively modest effect of relatively higher LA intake on body fat distribution could have an impact on metabolic health in a long-term perspective. Such speculation is supported by several trials showing that increasing dietary LA compared with PA is less obesogenic [5, 7, 12].

Whether different types of dietary fat can modulate total body fat content or adipose tissue distribution in humans is important for public health, and the mechanisms warrant further study. Although the results are in line with previous interventional data including younger subjects, the observed associations need to be confirmed in other populations and age groups before being extrapolated to the general population.

## Conclusions

The current findings are in accordance with recent data from randomized trials [5] and show that LA, primarily reflecting dietary n-6 PUFA intake, was negatively associated with VAT and SAT volumes, as well as with total body fat, trunk fat, leg fat and percent body fat. These results are also of metabolic interest since VAT and trunk fat were the depots most strongly associated with insulin resistance in this population, and visceral obesity predisposes individuals to cardiometabolic disease. Thus, the potential role of dietary fat quality in preventing excess VAT accumulation and insulin resistance in different populations warrants further investigation.

## Abbreviations

ALA: Alpha-linolenic acid
ALDH1A1: Aldehyde dehydrogenase 1 family member A1
BMI: Body mass index
CE: Cholesterol ester
D5D: Delta-5 desaturase
D6D: Delta-6 desaturase
DHA: Docosahexaenoic acid
DXA: Dual energy X-ray absorptiometry
EPA: Eicosapentaenoic acid
HOMA-IR: Homeostasis model assessment insulin resistance
IQR: Interquartile range
LA: Linoleic acid
MRI: Magnetic resonance imaging
MRS: Magnetic resonance spectroscopy
n-3: Omega-3
n-6: Omega-6
PA: Palmitic acid
PUFA: Polyunsaturated fatty acid
SAT: Subcutaneous adipose tissue
SCD: Stearoyl-coenzyme A desaturase
SD: Standard deviation
SFA: Saturated fatty acid
TLC: Thin layer chromatography
VAT: Visceral adipose tissue
